# Efficacy of endoscopic retrograde cholangiopancreatography in familial adenomatous polyposis patients after duodenectomy

**DOI:** 10.1002/deo2.85

**Published:** 2022-01-06

**Authors:** Ravi S. Shah, Neal Mehta, Carol A. Burke, Gautam Mankaney, Tyler Stevens, Toms Augustin, Matthew R. Walsh, Amit Bhatt

**Affiliations:** ^1^ Department of Gastroenterology, Hepatology, and Nutrition Cleveland Clinic Cleveland USA; ^2^ Department of Hepatobiliary Surgery Cleveland Clinic Cleveland USA

**Keywords:** adenomatous polyposis coli, cholangiopancreatography, endoscopic retrograde, endoscopy, gastrointestinal, pancreaticoduodenectomy, pancreatitis

## Abstract

**Objectives:**

Familial adenomatous polyposis (FAP) patients with Spigelman stage IV polyposis should be considered for prophylactic duodenectomy. Post‐surgical pancreaticobiliary complications occur and may require management via endoscopic retrograde cholangiopancreatography (ERCP). We aimed to assess the success and adverse events of ERCP in FAP patients after pancreas‐sparing duodenectomy (PSD) and pancreaticoduodenectomy (PD).

**Methods:**

A retrospective review of FAP patients who underwent ERCP after PSD or PD from 1992 to 2020 at a quaternary referral center was completed. The technical success of ERCP was defined as the ability to identify the anastomosis and cannulate the duct. Post‐procedural adverse events were defined by bleeding, perforation, pancreatitis, or cholangitis. Clinical outcomes included the need for surgical intervention and recurrent pancreatitis after ERCP were assessed.

**Results:**

Of 84 FAP patients with duodenectomy, 12 patients with PSD and two patients with PD underwent 17 ERCPs for pancreatic indications and five for biliary indications. The technical success of ERCP in patients with PSD and a single neoampullary complex for pancreatic (*n* = 6) and biliary (*n* = 5) indications was 100% but for those with PD (*n* = 2) or PSD reconstruction with pancreatic divisum or separate anastomoses (*n* = 3), it was 0%. Surgical intervention was required in 50% of patients with technically failed ERCP after PSD (2/4) and PD (1/2). There were no adverse events.

**Conclusions:**

ERCP is expected to be therapeutically successful for biliary complications following PSD. Assessment and potential therapy for pancreatitis post‐PSD are best in the setting of a single neo‐ampullary complex rather than in PD or PSD with pancreatic divisum.

## INTRODUCTION

Duodenal cancer is the second most common malignancy in patients with familial adenomatous polyposis (FAP) with a cumulative risk of 4%–10% by the age of 60.^1^, ^2^ Duodenal cancer arises from duodenal adenomas, often in association with advanced Spigelman stage of duodenal polyposis.^3^, ^4^, ^5^ In patients with Spigelman stage IV polyposis, the cumulative 10‐year risk of duodenal cancer is estimated to be 36% and therefore prophylactic duodenectomy should be considered.^1^, ^2^, ^3^, ^4^, ^5^

**FIGURE 1:**
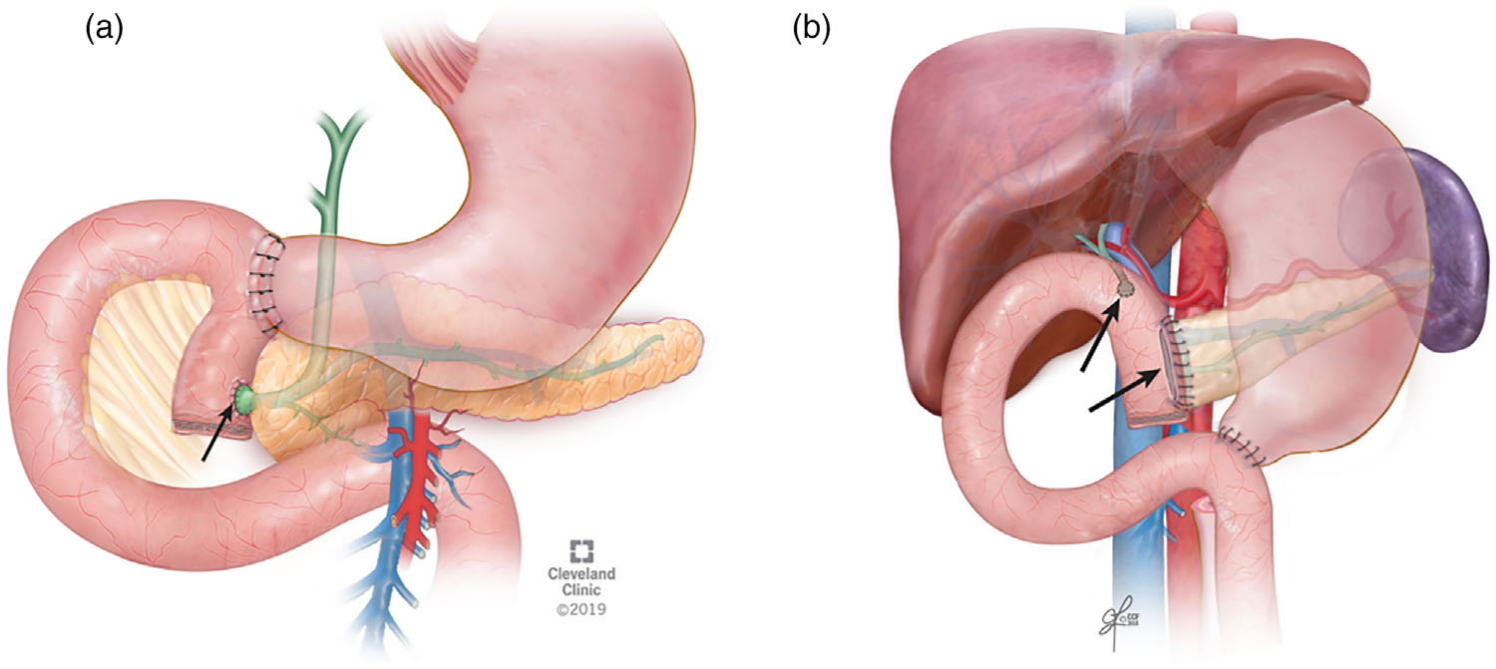
(a) Pancreas‐sparing duodenectomy: single anastomosis (Arrow) and (b) Pancreaticoduodenectomy: separate biliojejunal and pancreaticojejunal anastomoses (Arrows)

**FIGURE 2:**
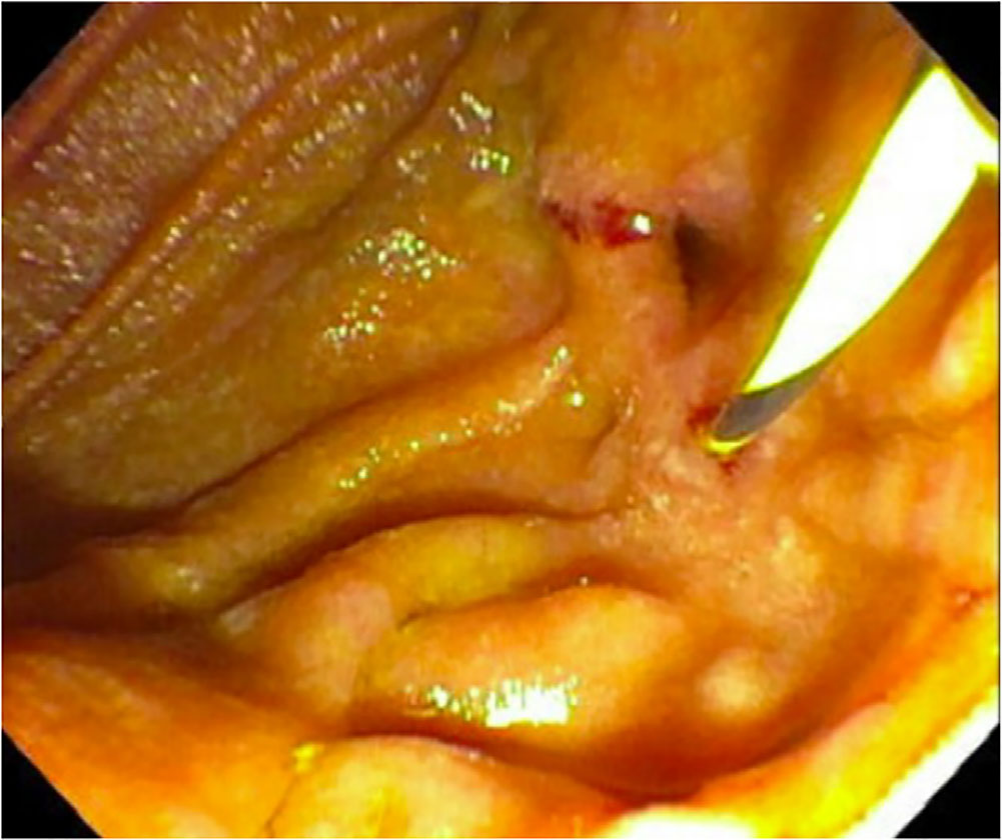
Pancreas‐sparing duodenectomy with neo‐ampullary complex in the neo‐jejunum, guidewire in the pancreatic orifice, biliary orifice seen above

Prophylactic surgery to prevent duodenal cancer in patients with advanced‐stage duodenal polyposis includes pancreas‐sparing duodenectomy (PSD) or pancreaticoduodenectomy (PD) with or without a pylorus‐sparing approach (Figure 1).^6^, ^7^, ^8^, ^9^, ^10^, ^11^, ^12^, ^13^, ^14^, ^15^, ^16^, ^17^, ^18^ PSD involves resection of the duodenum with resection of the native ampulla and the creation of two anastomoses, an end‐to‐side duodenal stump to the jejunal anastomosis and a single‐layer anastomosis between the pancreatic duct and bile duct and the advanced loop of jejunum thus recreating a neo‐ampulla (Figures 1a and 2). PD results in resection of the duodenum and pancreatic head with reconstruction requiring three anastomoses. First, a gastric‐or‐duodenal to jejunal anastomosis is created which leaves two limbs, an afferent jejunal limb that is used to create the pancreaticojejunostomy (PJ) and the hepaticojejunostomy and an efferent jejunal limb that restores intestinal continuity (Figure 1b).

Important considerations of the operation type chosen are endoscopic access to the postsurgical neo‐duodenum as the prevalence of duodenal bulb and jejunal polyposis can be as high as 80% and postsurgical adverse events can arise.^6^, ^7^, ^8^, ^9^, ^10^, ^11^, ^12^, ^13^, ^14^, ^15^, ^16^, ^17^, ^18^, ^19^, ^20^, ^21^ Endoscopic access in PSD compared to PD anatomy is facilitated by the lack of an afferent limb and a single ductal anastomosis (Figures 1 and 2). Complications of duodenectomy which may be managed endoscopically include anastomotic strictures, recurrent pancreatitis, adenomatous involvement of the neo‐papilla, or cholangitis.^20^

The outcomes of ERCP after PD in non‐FAP patients have been reported and success is limited by access to the ductal anastomoses.^22^, ^23^ Neo‐duodenal polyposis in FAP patients may further propagate this challenge.^6^ Although few reports of ERCP after duodenectomy in FAP patients exist, little is known regarding the outcomes of this approach.^14^, ^21^, ^24^ Therefore, we sought to evaluate the technical success of ERCP in patients with FAP who have undergone PSD or PD and subsequent clinical outcomes.

## METHODS

This is an institutional review board‐approved, single‐center, cohort study at the Cleveland Clinic including adult patients within the David G. Jagelman Inherited Colon Cancer Registries in the Sanford R. Weiss MD Center for Hereditary Colorectal Cancer. Patients diagnosed with FAP who underwent PSD or PD with at least 1 year of follow‐up, between years 1992 and 2019, and an ERCP (TJF‐Q160F/VF and TJF‐Q180V duodenoscopes [Olympus, Center Valley, PA, USA] or PCF‐H190DL and PCF‐H190L [Olympus]) for pancreaticobiliary indications were included. ERCPs, solely performed for removal of a stent that was previously placed by ERCP or intraoperatively, were excluded. Demographics, surgical details including indication for the operation, time from surgery to ERCP, and ERCP related factors including indication, technical success, adverse events, and clinical outcomes were collected from medical records. Technical success was defined by the ability to complete the intervention, including intubation of the afferent limb in PD anatomy, identification of the PJ and/or biliojejunal anastomoses, and duct cannulation. Adverse events were defined by perforation, immediate bleeding (intraprocedural bleed or hemoglobin (Hgb) drop within 24 h after procedure), delayed bleeding (post‐procedural Hgb drop within 2 weeks after the procedure), post‐ERCP pancreatitis (new/worsening abdominal pain, new/prolonged hospitalization for ≥2 days, and serum lipase/amylase >3x upper limit of normal, measured 24 h post‐procedurally), or cholangitis.^25^ Clinical outcomes were defined by the ability of ERCP to identify and/or resolve the etiology of the pancreaticobiliary complication, the number of ERCPs required to resolve the complication, the need for surgical revision, and recurrent pancreatitis. The primary outcome of this study was to determine the technical success of ERCP in FAP patients who have undergone duodenectomy and describe their subsequent clinical outcomes. Secondary outcomes included adverse event rates of ERCP.

Descriptive statistics are used to present demographics, ERCP and surgical details, technical success, adverse events, and clinical outcomes. Data are presented using frequency (*N*/*N*, %), mean +/‐ standard deviation (SD), and median (25th, 75th; %).

## RESULTS

### Patient, surgical, and procedural characteristics

**FIGURE 3:**
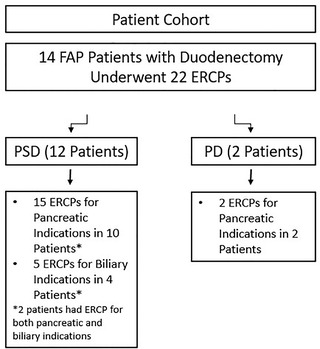
Patient cohort

Eighty‐four patients with FAP who underwent duodenectomy and had available follow‐up were identified. Of these, 14 (16.7%) patients (12 PSD, two PD) underwent 22 post‐surgical ERCPs (17 for pancreatic indications in 10 PSD and two PD, five for biliary indications in four PSD) (Figure 3). The mean age at the time of duodenectomy was 46.1 +/‐ 8.3 years and 57.1% of patients were male. The mean length of follow‐up for these patients was 13.9 +/‐ 5.2 years. The indication for PSD in all patients was Spigelman stage IV duodenal polyposis and was duodenal adenocarcinoma for the two patients that underwent PD (Table 1).

**TABLE 1:** Characteristics of familial adenomatous polyposis (FAP) patients who underwent pancreas‐sparing duodenectomy (PSD) or pancreaticoduodenectomy (PD) and endoscopic retrograde cholangiopancreatography (ERCP)

**Total patients**	***N* = 14**
Age at duodenectomy (years +/‐ SD)	46.1 +/‐ 8.3
Male	8 (57.1%)
PSD	12 (85.7%)
Indication: Spigelman stage IV polyposis, *N* (%)	12 (100%)
PD	2 (14.3%)
Indication: duodenal malignancy, *N* (%)	2 (100%)
Time from surgery to ERCP (years +/‐ SD)	7.0 +/‐ 4. 8
Total ERCPs	22
ERCP for pancreatic indications (PSD and PD) ERCP in ten PSD patients^a^ ERCP in two PD patients	17 (77.3%) 15 2
Pancreatitis	15 (88.2%)
Pancreatic duct leak	2 (11.8%)
ERCP for biliary indications (PSD only) ERCP in four PSD patients^a^	5 (22.7%) 5
Biliojejunal polyposis	1 (20%)
Choledocholithiasis	2 (40%)
Intrahepatic biliary dilation	2 (40%)
Procedural duration (min +/‐ SD)	54.8 +/‐ 27
Adverse events, % (*n/n*) Post‐ERCP pancreatitis Perforations Bleeding Post‐ERCP cholangitis	0 0 0 0

^a^ Two PSD patients had ERCP for both pancreatic and biliary indications

The median time between duodenectomy and ERCP was 7.0 +/‐ 4.8 years. Overall technical success in post‐PSD and PD anatomy was achieved in 14/22 (63.6%) ERCPs including all five (100%) of ERCPs done for biliary indications and 9/17 (52.9%) done for pancreatic indications (Table 2). There were no adverse events, including perforations, post‐procedural bleeding, or post‐ERCP pancreatitis (Table 1).

**TABLE 2:** Technical success of endoscopic retrograde cholangiopancreatography (ERCP) and identified etiologies

Length of follow‐up after ERCP (years +/‐ SD)	6.1 +/‐ 5.1
Total number of ERCPs per patient	One in seven patients Two in six patients Three in one patient
Overall technical Success of ERCP, % (*n/n*)	63.6 (14/22)
Technical success of ERCP for biliary indications % (*n/n*)	100 (5/5)
Etiologies of biliary indications in patients with successful ERCPs: Identification of polyposis involving biliojejunal anastomosis, % (*n/n*) Resolution of choledocholithiasis, % (*n/n*) Dilation of biliojejunal stenosis, % (*n/n*)	*N* = 4 50 (2/4) 25 (1/4) 25 (1/4)
Technical success of ERCP for pancreatic indications, % (*n/n*) Identification of the PJ anastomosis Cannulation of the PJ anastomosis Balloon dilation of PJ anastomosis Pancreatic duct stent placement	52.9 (9/17) 58.8 (10/17) 90 (9/10) 35.3 (6/17) 23.5 (4/17)
Etiologies of pancreatic indications in patients with successful ERCPs:	*N* = 6
No etiology identified	50 (3/6)
Etiology identified	50 (3/6)
Pancreatic duct leak Pancreatic duct stricture PJ anastomotic stricture	16.7 (1/6) 50 (3/6) 16.7 (1/6)

Abbreviation: PJ, pancreaticojejunostomy.

### PD: Technical success and clinical outcomes of ERCP for pancreatic indications

**FIGURE 4:**
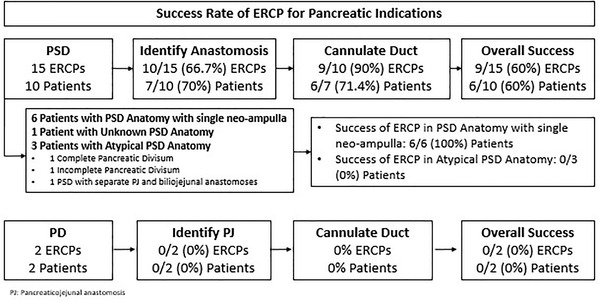
Success rate of endoscopic retrograde cholangiopancreatography (ERCP) for pancreatic indications

Two patients underwent pylorus‐preserving PD for duodenal adenocarcinoma and later developed pancreatitis with a dilated duct on cross‐sectional imaging requiring ERCP. The technical success of ERCP was 0/2 (0%), and the cause of failed ERCP was the inability to identify the PJ anastomosis in both patients (Figure 4).

### PSD: Technical success and clinical outcomes of ERCP for pancreatic indications

In a total of 10 patients with PSD anatomy, 15 ERCPs were performed for pancreatic indications, 13 for pancreatitis in nine patients, and two for pancreatic duct leak due to necrotizing pancreatitis in one patient (Figure 3). Five patients underwent two ERCPs, while the remainder each had one procedure performed.

**FIGURE 5:**
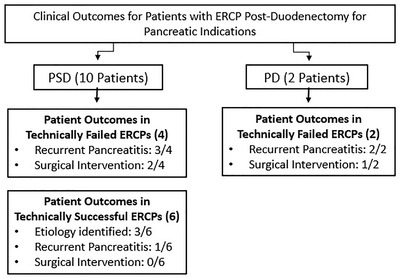
Clinical outcomes for patients with endoscopic retrograde cholangiopancreatography (ERCP) post‐duodenectomy for pancreatic indications

Technical success of ERCP in PSD patients was achieved in 6/10 (60%) patients. All six of these patients had PSD anatomy with a single ampullary complex (Figure 4). Note that, 3/6 (50%) of these patients had an etiology identified and/or resolved by ERCP, including stenosis of the PJ anastomosis (1/6), stricturing of the pancreatic duct (3/6), and pancreatic duct leak (1/6) (Table 2), and 2/6 patients (33.3%) required two ERCPs to achieve clinical success. Aside from the patient with pancreatic duct leak, none of the other patients had imaging prior to ERCP. In 3/6 (50%) of the remaining patients with technically successful procedures, ERCP did not reveal any abnormalities and therefore etiologies other than those related to surgery were considered. Only 1/6 of these patients with technically successful ERCPs developed recurrent pancreatitis over a follow‐up of 6.1 +/‐ 5.1 years. None of these patients required surgical intervention for further management (Figure 5).

Of the four (40%) patients with technically failed ERCPs post‐PSD, three (75%) patients did not have a single neo‐ampullary reconstruction, with two having a dominant dorsal duct, and one with separate pancreatic and biliary orifices due to a distal biliary stricture (Figure 4). Reasons for failed ERCPs in post‐PSD anatomy included the inability to identify the anastomosis (3/4 patients) or cannulate the pancreatic duct (1/4 patients) (Figure 4). All four of these patients had imaging prior to attempted ERCP and 3/4 of patients had a dilated pancreatic duct. In one of these patients, ERCP confirmed pancreatic duct dilation but failed due to the inability to deeply access the pancreatic duct. Three patients developed recurrent pancreatitis over a follow‐up period of 7.1 years. Two (50%) patients required surgery to further manage recurrent pancreatitis (Figure 5).

**FIGURE 6:**
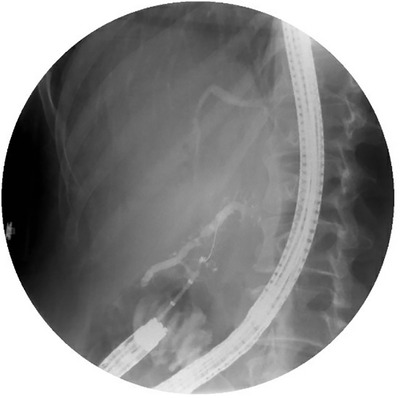
Incomplete pancreatic divisum in pancreas‐sparing duodenectomy

Surgical details of the four patients above were examined. The first patient was known to have pancreatic divisum at the time of PSD and underwent reimplantation of the ventral and dorsal ducts. The patient developed recurrent pancreatitis and ultimately underwent a pancreatic head resection with pancreatojejunostomy, hepaticojejunostomy, and duodenojejunostomy after two failed attempts by two endoscopists to find either of the reimplanted pancreatic ductal anastomoses. The patient continued to have recurrent pancreatitis requiring hospitalizations despite surgical revision over a 7‐year follow‐up period. The etiology of recurrent pancreatitis was attributed to various possible etiologies, including reflux of enteric contents via a patent anastomosis in the setting of multifocal desmoids, alcohol use, smoking, and sulindac, and did not require further endoscopic or surgical intervention after risk factor management. The second patient was found to have an incomplete pancreatic divisum with a dorsal dominant pancreatic duct at the time of ERCP after an episode of pancreatitis (Figure 6). ERCP was unsuccessful due to the inability to cannulate the main pancreatic duct, and the patient underwent a Puestow procedure (lateral PJ) to manage recurrent pancreatitis. Despite undergoing this surgical revision, the patient developed recurrent pancreatitis over a one‐year follow‐up period. In patients with pancreatic divisum, therapeutic intervention to the minor duodenal papilla was limited primarily due to the inability to identify either of the major or minor papillary openings. The third patient underwent PSD with a separate biliojejunal anastomosis from the PJ anastomoses, similar to the anatomy of PD. Two failed ERCPs by three different endoscopists were due to the inability to identify the PJ anastomosis. The final patient with a failed ERCP due to the inability to identify the ampullary complex had an unknown surgical history.

### PSD: Technical success and clinical outcomes of ERCP for biliary indications

Five ERCPs were performed for biliary indications in four patients with PSD anatomy. One patient required two procedures, and three patients required one ERCP each.

The technical and clinical success rate for ERCP for biliary indications in PSD anatomy was 5/5 (100%). The biliary indications for ERCP varied (Table 2). In two patients, ERCP was performed to evaluate polyposis involvement of the biliojejunal anastomosis in which cholangioscopy revealed low‐grade adenomatous involvement of the bile duct. Another patient had ERCPs done for two different episodes of choledocholithiasis approximately two years apart. The last patient had ERCP to evaluate intrahepatic biliary dilation, seen on MRCP. ERCP revealed biliojejunal anastomotic stenosis and associated common and intrahepatic bile duct dilation. None of these patients had endoscopic adverse events, including immediate or delayed postprocedural bleeding, perforation, or post‐ERCP pancreatitis.

## DISCUSSION

The results of this study suggest that ERCP is technically and clinically successful in all patients with pancreatic indications in PSD anatomy where there is a single neo‐ampulla, but unsuccessful in patients with dominant dorsal ducts or multiple pancreaticobiliary anastomoses. ERCP is technically and clinically successful for biliary complications after PSD.

PSD is advantageous compared to PD for the management of FAP‐associated advanced duodenal polyposis. A single ampullary complex is anastomosed to the duodenal wall in PSD (Figures 1a and 2) rather than the creation of two separate PJ and biliojejunal anastomoses in PD (Figure 1b), which creates an afferent limb that is difficult to survey and does not lend itself to therapeutic interventions remote from the surgery. There are limitations to performing PSD with a single neo‐ampullary complex. One disadvantage is a neo‐duodenal transposition in PSD may be limited by desmoid tumors causing bowel fixation, but that is equally challenging in the reconstruction for PD. Additionally, as suggested by our results, a single ampullary anastomosis may be limited by the presence of a distal biliary stricture or divisum anatomy. PD rather than PSD is required in patients with invasive duodenal malignancy that carries an increased risk of lymph node metastasis or widespread metastatic disease. Given the lack of long‐term data, PSD is not considered an alternate operation to PD in the management of duodenal cancer.^15^, ^20^ In our experience, reconstruction with a neo‐ampulla after a PSD leads to an optimal outcome of assessing for post‐surgical complications.

The success rate of ERCP in patients with a single neo‐ampullary complex was 100% in this series, and it is the only anatomical configuration possible. Most neo‐ampullary complexes in PSD allow for distinct cannulation of the biliary and pancreatic duct orifice as seen in Figure 2. During PSD, before the ampullary‐jejunostomy is created, the exposed ampulla undergoes a sphincteroplasty and septoplasty allowing for the creation of the anastomosis with separate ducts. The challenge is identifying the ampulla due to the presence of polyposis, but once identified, selective biliary and pancreatic cannulation can be easily performed. Of the patients that did not have a single neo‐ampullary complex and failed post‐surgical ERCP, two patients had pancreatic divisum and one patient had a distal biliary stricture requiring a separate anastomosis. As a result, we no longer perform PSD in patients with pancreatic divisum and attempt to identify these patients preoperatively or intra‐operatively which results in conversion to PD. In our patients, the therapeutic intervention was limited by the inability to identify the orifices. This underlines the importance of the endoscopist's awareness of these patients’ postsurgical anatomy to better guide the endoscopic approach.

Pancreatitis has been a reported adverse event after PSD at rates of 8%–22%.^20^, ^26^ Pancreatitis due to stricture of the PJ anastomosis or pancreatic duct following PD or PSD can be delayed from the time of surgery, as reflected by our median time from duodenectomy to ERCP of 7.0 +/‐ 4.8 years, and highlights the need for long‐term consideration of this outcome.^20^, ^26^ In our study, the etiologies of pancreatitis elucidated by successful ERCPs in PSD anatomy included pancreatic ductal or PJ anastomotic stricture. The strategy for the management of anastomotic pancreatic ductal stricture was at the discretion of the endoscopist. Generally, for pancreatic strictures, a combination of balloon dilation and stent placement (7 Fr) was performed with upsizing of stents until resolution of stricture. Pancreatic duct stents were evaluated for upsizing, replacement, or removal after 4–6 weeks of initial placement. Aside from stricture, the other etiologic consideration of pancreatic duct outflow obstruction is a recurrence of adenoma at the neo‐papillary anastomosis although this is distinctly uncommon in our experience. Given that 50% of technically successful ERCPs were unable to identify an etiology of pancreatitis, other etiologies must be considered in this patient population.

Unlike prior studies, etiologies of recurrent pancreatitis were not related to PJ anastomotic or ductal stricture in 50% of our patients with technically successful ERCPs, perhaps suggesting backwash of biliary‐enteric contents into an otherwise normal pancreatic duct or a non‐post‐surgical etiology.^24^, ^26^, ^27^ Pre‐procedural imaging (i.e., MRCP or computed tomography pancreas) may identify patients who clinically benefit from an ERCP intervention by delineating etiologies such as strictures. Possible non‐anatomical etiologies that are unique to FAP patients are sulindac use and endoscopic biopsy of the neo‐papilla, although exceedingly rare.^28^, ^29^, ^30^, ^31^, ^32^ Idiopathic recurrent pancreatitis in FAP has been reported and may be a manifestation of the APC gene mutation.^28^, ^29^ The long duration between duodenectomy and ERCP, especially in those lacking evidence of stricture, may support the hypothesis that FAP patients have an inherent predisposition to developing pancreatitis. After considering possible non‐anatomic etiologies of pancreatitis, ERCP is a tool to investigate anatomic etiologies of post‐duodenectomy pancreatitis in FAP patients.

All ERCPs successfully intubated the limb harboring the orifices. The use of a side‐viewing scope in PSD allows for more control over ERCP delivery and cannulation compared to a long‐wire system without elevator control used in ERCP with a PCF scope utilized in PD anatomy. All ERCPs performed at our institution in PD utilized a PCF (PCF‐H190DL and PCF‐H190L [Olympus]). This is the preference at our institution because the anastomosis is within endoscopic reach and the endoscopists are accustomed to the ERCP equipment. It should be noted that a balloon enteroscopy is an important tool in patients with longer afferent limbs or adhesions causing looping to access either ductal anastomosis. Balloon‐assisted enteroscopy is known to be successful for biliary indications after PD, however, has been reported to have lower success for pancreatic indications ranging from 38%–71% primarily due to pancreatic duct identification and cannulation.^33^, ^34^, ^35^, ^36^

Identification of the PJ anastomosis, despite temporary postsurgical pancreatic duct stent or suture placement, remains difficult in patients presenting with late post‐surgical pancreatitis. The reason for the inability to find the PJ anastomosis is unclear, however, our findings are consistent with previous studies reporting a ≤50% success of identifying the PJ anastomosis in non‐FAP patients with PD.^22^, ^23^ Hypothesized possibilities include PJ anastomotic endothelialization, peri‐anastomotic edema, and polyposis.^20^, ^21^ Technologic advancements in endoscopy have curbed the challenges of managing patients with post‐surgical anatomy.

When conventional pancreatic duct access fails, endoscopic ultrasound (EUS)‐guided access may achieve technical success but comes with added risks. A systematic review comparing EUS‐guided pancreatic duct drainage versus ERCP pancreatic duct drainage determined that a EUS‐guided approach had superior rates of pancreatic duct opacification (87% vs. 30%; *p* < 0.001), cannulation success (79 % vs. 26 %; *p* < 0.001), and stent placement (72 % vs. 20 %; *p* < 0.001).^33^ The rates of adverse events in a systematic review and meta‐analysis reports rates of more serious complications of EUS‐pancreatic duct drainage to be acute pancreatitis 6.6 % (95 % confidence interval [CI], 4.5–9.4), bleeding 4.1 % (95 % CI, 2.7–6.2), perforation and/or pneumoperitoneum 3.1 % (95 % CI, 1.9–5), pancreatic leak and/or pancreatic fluid collection 2.3 % (95 % CI, 1.4–4), and infection 2.8 % (95 % CI, 1.7–4.6).^37^ Mortality has been reported with EUS‐guided biliary drainage and should be acknowledged as a potential adverse event.^38^, ^39^

The retrospective nature of the series limits some interpretation of the results. The small sample size does not allow the findings to be strongly conclusive or generalizable, however provides insight into a rare patient population. Generalizability is limited by inter‐endoscopic variability as ERCP in postsurgical anatomy is technically challenging and often referred to major tertiary centers. Our patient cohort consists of an uneven sample size as PSD was more frequently performed over PD in FAP patients with advanced‐stage duodenal polyposis.

ERCP appears to be useful in managing pancreatitis secondary to PJ anastomosis or pancreatic duct stricture in FAP patients with PSD anatomy without carrying significant procedural adverse events but has limited utility in those with pancreatic divisum or distinct PJ and biliojejunal anastomoses (i.e., PD). Endoscopic innovation, utilizing EUS‐guided pancreatic ductal access, in managing patients with unidentifiable PJ anastomosis or failed pancreatic duct cannulation is required.

## CONFLICT OF INTERESTS

Carol A. Burke has no relevant disclosures but has research support from Janssen Pharmaceuticals, Ferring Pharmaceuticals, and Cancer Prevention Pharmaceuticals and consulting fees from Freenome, Ferring Pharmaceuticals, and SLA Pharma. Amit Bhatt has no relevant disclosures but has royalties from Medtronics and consulting fees from Boston Scientific, Lumendi, Steris, Intuitive. All other authors do not have any conflict of interest.

## ETHICS STATEMENT

The research protocol was approved by the Cleveland Clinic Institutional Review Board.

## FUNDING INFORMATION

None.
